# Dynamics and Distribution of the Invasive Mosquito *Aedes koreicus* in a Temperate European City

**DOI:** 10.3390/ijerph17082728

**Published:** 2020-04-15

**Authors:** Kornélia Kurucz, Mattia Manica, Luca Delucchi, Gábor Kemenesi, Giovanni Marini

**Affiliations:** 1 Institute of Biology, Faculty of Sciences, University of Pécs, H-7624 Pécs, Hungary; 2 Department of Biodiversity and Molecular Ecology, Research and Innovation Centre, Fondazione Edmund Mach, 38010 San Michele all’Adige, Italy

**Keywords:** mosquito surveillance, mathematical model, invasive species, Culicidae, urban area

## Abstract

*Aedes koreicus* is a mosquito species native to Asia that has recently successfully invaded new areas in several European countries. Here, we provide important data on *Ae. koreicus* establishment in Pécs (Southern Hungary). Mosquito surveillance was carried out weekly between 2016 and 2019 at 10 different sites located throughout the city from May to September. We conducted a statistical analysis to evaluate the most important abiotic factors driving *Ae. koreicus* abundance. We then calibrated a previously developed temperature-dependent mathematical model to the recorded captures to evaluate mosquito abundance in the study area. We found that too high summer temperatures negatively affect mosquito abundance. The model accurately replicated the observed capture patterns, providing an estimate of *Ae. koreicus* density for each breeding season, which we interpolated to map *Ae. koreicus* abundance throughout Pécs. We found a negative correlation between mosquito captures and human density, suggesting that *Ae. koreicus* does not necessarily require humans for its blood meals. Our study provides a successful application of a previously published mathematical model to investigate *Ae. koreicus* population dynamics, proving its suitability for future studies, also within an epidemiological framework.

## 1. Introduction

Many invasion events by different alien mosquito species have occurred in Europe in recent decades [1]. Therefore, entomological surveillance is critical to understand mosquito population invasion processes and their potential impact on human health. In particular, *Aedes* species are of greater concern due to their competence as vectors of several pathogens such as, for instance, dengue, chikungunya, and Zika viruses [2]. Improvement in surveillance systems allows better detection of the introduction of invasive *Aedes* species [3,4,5,6]. However, in the face of the new challenges posed by global environmental change and insecticide resistance, there is the need to develop a novel approach to mosquito control based on the One Health perspective rather than the insecticide-only approach [7,8]. For instance, in Italy, West Nile virus is managed through an annually revised plan aiming to reduce the risk of transmission to humans by early detection of viral circulation through surveillance not only in mosquitoes but also in birds and horses [9].

Among *Aedes* invasive mosquito species, the most successful one is *Ae. albopictus* (Skuse, 1894), which is now widely spread in the southern part of the continent. In particular, it has established itself in the southern parts of Spain and France, Italy and most of the outer regions of Balkan Peninsula [10]. In 2008, *Ae. koreicus* (Edwards, 1917) was found in Europe (in Belgium) for the first time [11]. This species is native to Japan, China, South Korea, and Eastern Russia [12], but it has now established itself in several European countries, namely Italy, Germany, Belgium, and Hungary [10]. Because it showed a highly invasive potential in the last decade, increased attention should focus on this species to reveal its current and exact distribution range in Europe.

In Hungary, it was found for the first time in 2016 in Pécs, a medium-sized city located in the southwestern part of the country, close to the country’s border with Croatia [13]. Since then, the species has successfully established in the city and has become a dominant part of the native mosquito fauna [14]. Its colonization of urban areas, daytime biting, and opportunistic feeding behavior increase the likelihood that this species will pose a serious public health risk [12,15]. The importance of this new invasive species with regard to human and veterinary health is indubitable. It can transmit the heartworm *Dirofilaria immitis* [16], endemic in Europe, and could be involved in chikungunya virus transmission as well [17], which caused several outbreaks in the last decade on the continent. In particular, so far, two large outbreaks in Italy (2007 and 2017) and three smaller outbreaks in France (2010, 2014, and 2017) have been recorded, with more than 500 people infected in total [18]. Gut microbiota, which can influence mosquitoes’ vector competence, has recently been studied in *Ae. koreicus* to find potential bacterial candidates that could be used in control strategies against this mosquito vector and its pathogens [19].

However, compared to *Ae. albopictus*, limited knowledge is available regarding the potential for the establishment and spread of this species in Europe. Most of the information referring to the biology and behavior of *Ae. koreicus* (including its habitat preferences, feeding behavior, and overwintering strategy) originated from its native territories [11,15,20]. Similarly to other mosquito species, in laboratory conditions, temperature was found to have a strong influence on the biology of *Ae. koreicus*, affecting, for instance, the developmental time and survival of immature stages and adult longevity [21]. In particular, temperatures ranging between 23 and 28 °C are favorable for this species’ development [21], explaining its recent success at establishing itself in new temperate European areas. 

In this study, we aimed to analyze the dynamics and distribution of *Ae. koreicus* within a temperate European city by providing new data on the species abundance in space and time in Pécs, Hungary. For this purpose, we applied a previously published mathematical model [21], which is explicitly driven by temperature, to replicate the observed mosquito dynamics and provide an estimate of *Ae. koreicus* density over the whole city. This kind of model has been widely used to investigate mosquito dynamics, also within an epidemiological framework [22,23,24,25], and is usually temperature-dependent as this abiotic factor plays a key role at shaping mosquito survival and development [21,26,27,28].

## 2. Materials and Methods

### 2.1. Study Area and Data Collection

**Figure 1:**
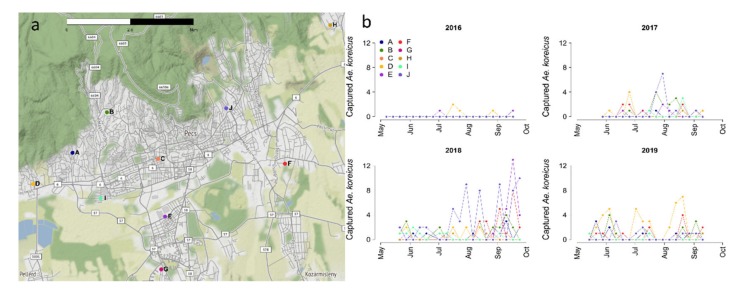
(**a**) Study area and traps (A–J) location. (**b**) Recorded number of trapped adult *Aedes koreicus* females per trap and year. Map data © OpenStreetMap contributors [31]. Numbers are presented in Table S1: Number of trapped Ae. koreicus for each trap and sampling date (Julian day of the year).

As shown in Figure 1, the surveillance was conducted in the city of Pécs (46°06′27.31” N, 18°12′24.17” E), the administrative and economic center of Baranya county in Southwest Hungary. The city covers an area of 162.8 km^2^ and has a total population of 142,873 [29]. The region is characterized by a temperate climate, with a mean annual precipitation of 672 mm and a mean annual temperature of 14 °C [30].

For mosquito monitoring, Heavy-Duty Encephalitis Vector Survey (EVS) Traps (Bioquip, Rancho Dominguez, CA, USA) were used at 10 sampling sites in an urban area (Figure 1a), with dry ice as the CO_2_ attractant. The sampling was carried out from 2016 to 2019 during the general mosquito season from May to September. Traps were operated once per week overnight (6:00 p.m.–6:00 a.m.), for a total of about 20 sampling sessions per year. The captured adult mosquitoes were identified morphologically on the species level [32,33] using a stereomicroscope. Then, abundance data per trap and per trapping session of female *Ae. koreicus* were used for further statistical analyses.

For each trap location, daily average temperature data were obtained from the gap-free Moderate Resolution Imaging Spectroradiometer (MODIS) Land Surface Temperature (LST) maps at a resolution of 250 m [34]. Temperature patterns for each trap and year are shown in Figure S1. We calculated the percentage of the different land cover classes contained inside a buffer of radius 250 m using the 2018 Corine dataset [35]. We identified 5 different classes covering the 10 buffers: “discontinuous urban fabric”, “industrial or commercial units”, “non-irrigated arable land”, “complex cultivation patterns”, and “land principally occupied by agriculture with significant areas of natural vegetation”. We defined as vegetation cover the proportion of land covered by the latter three categories. Human population data were collected from the Worldpop database [36] at about 100 m resolution over the considered study region (Figure S2).

### 2.2. Statistical and Mathematical Models

Because of the few adults recorded in 2016 (see results), we decided to carry out our quantitative analysis only starting from 2017. The relationship between socioenvironmental factors and temperature and *Ae. koreicus* abundance was investigated through nonparametric correlation tests and generalized linear models (GLMs) assuming a negative binomial distribution for the response variable. Response variable, denoted by Y, is the yearly total number of *Ae. koreicus* adult females collected in each sampling site. GLMs were used to assess the effect of temperature on mosquito abundance by alternatively considering as explanatory variable X two different averaging windows for LST, namely spring (April–May) and summer (June–August). The two models can therefore be represented by the following equations:

Y~NB (µ, θ)(1)

Log (µ) = a + bX(2)

E (Y) = µ, Var (Y) = µ + µ^2^/θ (3)

where NB indicates a negative binomial distribution of parameter µ (mean) and θ (dispersion parameter). Kendall correlation tests were performed to evaluate the association between human density and vegetation cover and Y, the yearly total number of *Ae. koreicus* adult females collected in each sampling site.

We then calibrated a previously published mathematical model, defined as a “population model” [21], to the weekly number of trapped mosquitoes averaged over the 10 collection sites. The model provides a daily estimate of *Ae. koreicus* dynamics for each population stage (eggs, larvae, pupae and adults) explicitly considering temperature, as it drives most of the developmental and survival rates. More specifically, such rates are estimated through temperature-dependent functions calibrated on the experiments presented in the same study, allowing also for some uncertainty [21]. The population model has two free parameters: the daily capture rate of adult mosquitoes α and a density-dependent scaling factor *K* driving the carrying capacity for the larval stages. α is assumed to be equal among different years, *K* is assumed to be year-specific. Thus, there are 4 parameters to estimate that form the set of unknown parameters Ψ, where $\Psi =\left\{\alpha ,K{\left(y\right)}_{y\in \left\{2017,2018,2019\right\}}\right\}$. The posterior distributions of Ψ were estimated by Markov Chain Monte Carlo (MCMC) sampling. The Poisson likelihood of the observed averaged weekly captures given model-predicted captures was multiplied across the 3 datasets (3 years) under study to provide the overall likelihood of observations:

(4) $$L=\overset{2019}{\underset{y=2017}{\prod }}\overset{M}{\underset{m=1}{\prod }}e^{-C\left(y,m,\psi \right)}\cdot \frac{C{\left(y,m,\psi \right)}^{n\left(m,y\right)}}{n\left(m,y\right)!}$$

where *y* and *m* run over the considered years and trapping sessions respectively, *M* is the total number of trapping sessions carried out during one year, *n*(*m,y*) is the observed number of *Ae. koreicus* adults averaged over the 10 traps, and *C*(*y,m*,Ψ) is the number of captures predicted by the population model with parameters Ψ. A comprehensive description of the model and its parameters, including their laboratory estimation, and additional details on the calibration technique can be found in [21].

Finally, by considering the results of the statistical analyses and of the entomological model, we mapped *Ae. koreicus* abundance throughout the study area. More specifically, we assumed that a trap on average covers an area Ω = π*r*^2^, where *r* is the average *Ae. koreicus* daily flight range (150 m as previously used in [21]). Consequently, the entomological model provides an estimate *M(t)* of mosquito abundance for Ω for any day *t*. So, if *A* is the area of the whole considered region presented in Figure 1a, then by multiplying *M(t)* for the ratio *A*/Ω, we can obtain an estimate of the total number of mosquitoes present in the city on day *t*. We then redistributed this value over each hectare of the study region through a standard universal kriging procedure using human density as a predictor, as we found a significant relationship between this quantity and the total number of trapped mosquitoes (see Results).

The statistical analyses, elaboration of model outcomes, and kriging interpolation were carried out in R v3.6.0 [37] using packages raster [38], automap [39], MASS [40].

## 3. Results

### 3.1. Entomological Collections

**Figure 2:**
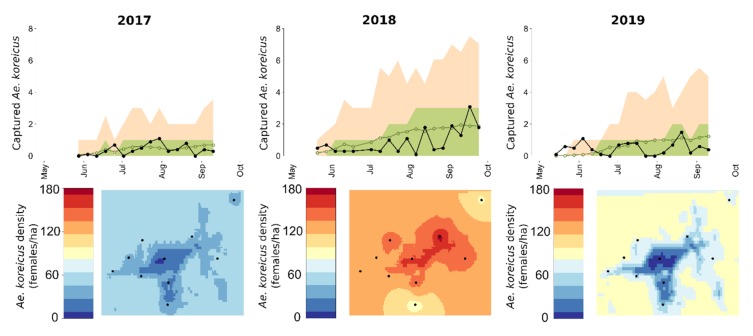
First row: 95% and 50% Credible Intervals (CI) (orange and green areas respectively) and average number (green line) of trapped *Ae. koreicus* females for each year predicted by the population model. Black line shows average of the recorded captures. Second row: number of *Ae. koreicus* adult females per hectare (kriging of the population model prediction) averaged between June and September for each year. Black dots represent the 10 trapping sites.

The total numbers of trapped female *Ae. koreicus* over all sites were 6, 61, 161, and 87 in 2016, 2017, 2018, and 2019, respectively. We observed substantial heterogeneity between traps, for instance, trap G captured in total only 1 adult in 2017, whereas trap J captured 80 specimens over the four considered years (Figure 1b). Due to the very few adults recorded in 2016, we decided to carry out our quantitative analysis only starting from 2017. As shown in Figure 2, the averaged captures were quite different between the three years; in 2017 and 2019, the population seemed rather stable during the season, whereas in 2018, it started to rise significantly toward the end of summer.

### 3.2. Modelling Results

As shown in Table 1, we found that spring temperature is not significantly associated with the total number of trapped *Ae. koreicus*, while conversely summer temperature had a negative significant effect on this quantity. For instance, according to the statistical model, a temperature shift from 23 to 26 °C corresponded on average to an 82% decrease in the number of trapped mosquitoes. Similarly, we found no significant association with vegetation cover, whereas the number of trapped mosquitoes was higher where human density was lower.

As reported in Figure 2 (first row), the population model fits the observed average weekly captures quite well. Considering the three modeled years (2017–2019), 94.2% (78.8%) of the observed captures lie within the 95% (50%) credible intervals (CIs) of model predictions. In particular, the population model is able to replicate the increasing abundance observed in 2018 at the end of the season.

The estimated capture rate α was on average 0.015 (95% CI 0.005–0.033), meaning that about 1.5% of the host-seeking females were captured in one day. As expected, the larval density dependent factor *K* varied between years, with higher values for 2018 (see Figure S3).

Mosquito city abundance was evaluated at a 100 m resolution by universal kriging considering human density as a predictor, since we found a significant relationship between these two quantities. As shown in Figure 2, we estimated a quite heterogeneous, both spatially and temporally, *Ae. koreicus* distribution over the city of Pécs. Average predicted summer density ranged between 8 and 57, 87 and 179, and 3 and 97 females per hectare in 2017, 2018, and 2019, respectively. The negative relationship with human density was more marked in 2017 and 2019, whereas for 2018, *Ae. koreicus* density was estimated to be larger and more homogeneous throughout the city, as expected, given the higher number of trapped mosquitoes.

## 4. Discussion

In this study, we provided new data on the invasion of *Ae. koreicus* in a temperate European country where the species was recently introduced and established. We calibrated a previously developed temperature-dependent population model [21] on the mosquito captures recorded in the area. Although the model was initially derived with data gathered in a different study region (Northern Italy), we found that it can accurately replicate the temporal dynamics observed in Pécs, Hungary. As previously noted [21], the model tends to overestimate *Ae. koreicus* abundance especially toward the end of the season, possibly due to the lack of a diapausing mechanism, a biological feature not yet investigated in detail for this species. We estimated a lower capture rate with respect that reported in [21] for BG traps (Biogents AG, Regensburg, Germany), suggesting that EVS CO_2_ traps deployed in Pécs might be less effective for capturing *Ae. koreicus*. In urban environments, the BG trap has a significantly better trapping efficacy compared to the EVS trap, including diversity of mosquito species and number of mosquitoes per trapping period [41]. Thus, the BG trap likely represents a better solution for general surveillance programs of adult mosquitoes in Europe.

We found a negative relationship between summer temperature and the number of trapped mosquitoes, implying that too warm conditions might decrease *Ae. koreicus* survival. Such association was observed in Northern Italy as well; during field collections carried out over three years at four different locations, the higher numbers of trapped mosquitoes were recorded in the coldest sites [21]. Breeding sites availability might be another crucial factor for *Ae. koreicus* abundance. According to our estimates (Figure S3), the density-dependent scaling factor driving the larval carrying capacity varied substantially between different years, possibly reflecting different numbers and types of breeding sites available for this species during different years.

Another important biological factor for population abundance might be competition for resources. For instance, a weak larval interspecific competition has been demonstrated under laboratory conditions between *Ae. albopictus* and *Ae. koreicus*, with a slight advantage for the former [42]. In the study area, *Ae. albopictus* is not present, but *Ae. vexans* is quite abundant (results not shown), and if the same asymmetrical competition occurs between these two species, this might help explain different abundances observed between years.

Although *Ae. koreicus* is known to be better adapted to urban environments than *Ae. japonicus* (Theobald, 1901) [11,12], our statistical analyses highlighted a slightly negative correlation between mosquito captures and human density. These results also suggested that *Ae. koreicus* can complete its life cycle feeding on animals other than humans, as already suggested by samplings in Northern Italy during which larvae of this species were found far from human settlings [43]. Preliminary blood meals analyses showed that, in the study area, this mosquito species might feed on humans as well [44]. However, since blood meal identification was carried out for only one specimen, a more extensive investigation is needed to provide a robust estimation of the feeding preference of this species.

The negative association we found between *Ae. koreicus* abundance and human density is reflected also in the interpolated maps (Figure 2) obtained for 2017 and 2019, whereas in 2018, *Ae. koreicus* density was estimated to be more uniform over the study area. Mosquitoes kriged densities are comparable to those obtained with the same population model in Northern Italy [21], suggesting a similar *Ae. koreicus* abundance between the two regions.

## 5. Conclusions

Hungary finds itself exposed to invasion from not only *Ae. koreicus* but also from *Ae. albopictus* and *Ae. japonicus*. *Ae. albopictus* has not invaded Pécs yet, but it is already present in some areas in the country [10]. *Ae. japonicus* was found in small villages along the main road from Pécs to the Croatian border (Drávaszabolcs), but not in the study area (Kurucz, personal communication). Enhanced entomological surveillance, such as the one deployed in Pécs, and clear guidelines and protocols for integrated vector management are thus urgently needed [45,46]. The present model is a first step toward the integrated management of *Aedes*-borne diseases as it takes advantage of entomological monitoring data to build a population abundance dynamics that could be used to inform disease transmission risk models [47]. However, the presence of high variability in trap captures as well as the limited number of observations currently limit the population model’s predictive performance as well as the robustness of the statistical associations estimated between mosquito abundance and eco-climatic variables. Therefore, results should be interpreted with caution and further investigated.

## Figures and Tables

**Table 1:** Results of the statistical analyses. Coefficients estimates for generalized linear models are at log scale.

Explanatory Variable	Statistical Test/Model	Coefficients Estimate	*p*-Value
Average spring temperature	Negative Binomial GLM	Intercept = –1.47	0.565 0.174
		Coef = 0.21	
Average summer temperature	Negative Binomial GLM	Intercept = 16.57	0.005 0.016
		Coef = –0.57	
Human population	Kendall correlation test	*τ* = –0.29	0.03
Vegetation cover	Kendall correlation test	*τ* = 0.09	0.49

## Supplementary Materials

The following are available online at https://www.mdpi.com/1660-4601/17/8/2728/s1, Figure S1: Land Surface Temperature for each trapping site (A–J), Figure S2: Human population density (number of human beings living in approximately one hectare). Black dots represent the mosquito trapping sites, Figure S3: Boxplots (median, quartiles and 95% quantiles) of the estimated distributions of the model free parameters. (a) α, the capture rate; (b) K, the larval density dependent factor, which is year dependent, Table S1: Number of trapped Ae. koreicus for each trap and sampling date (Julian day of the year).
