# Supplementary figures and images for: Dynamics and Distribution of the Invasive Mosquito Aedes koreicus in a Temperate European City
Source: Int J Environ Res Public Health. 2020 Apr 15;17(8):2728. doi: 10.3390/ijerph17082728 (PMC7216222; doi:10.3390/ijerph17082728)

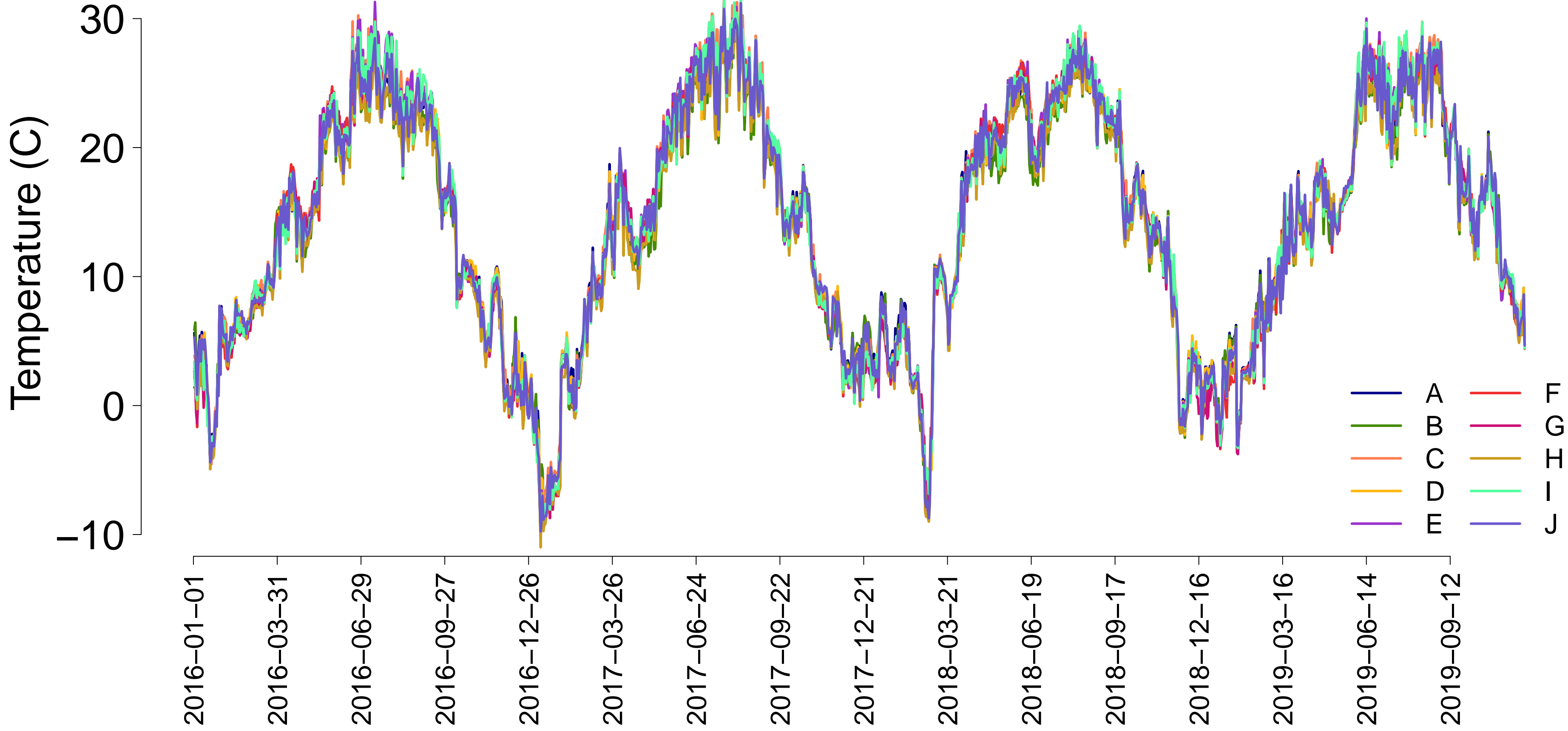

Supplement: Supplementary file 1 [file ijerph-17-02728-s001.zip › Figure S1.pdf]

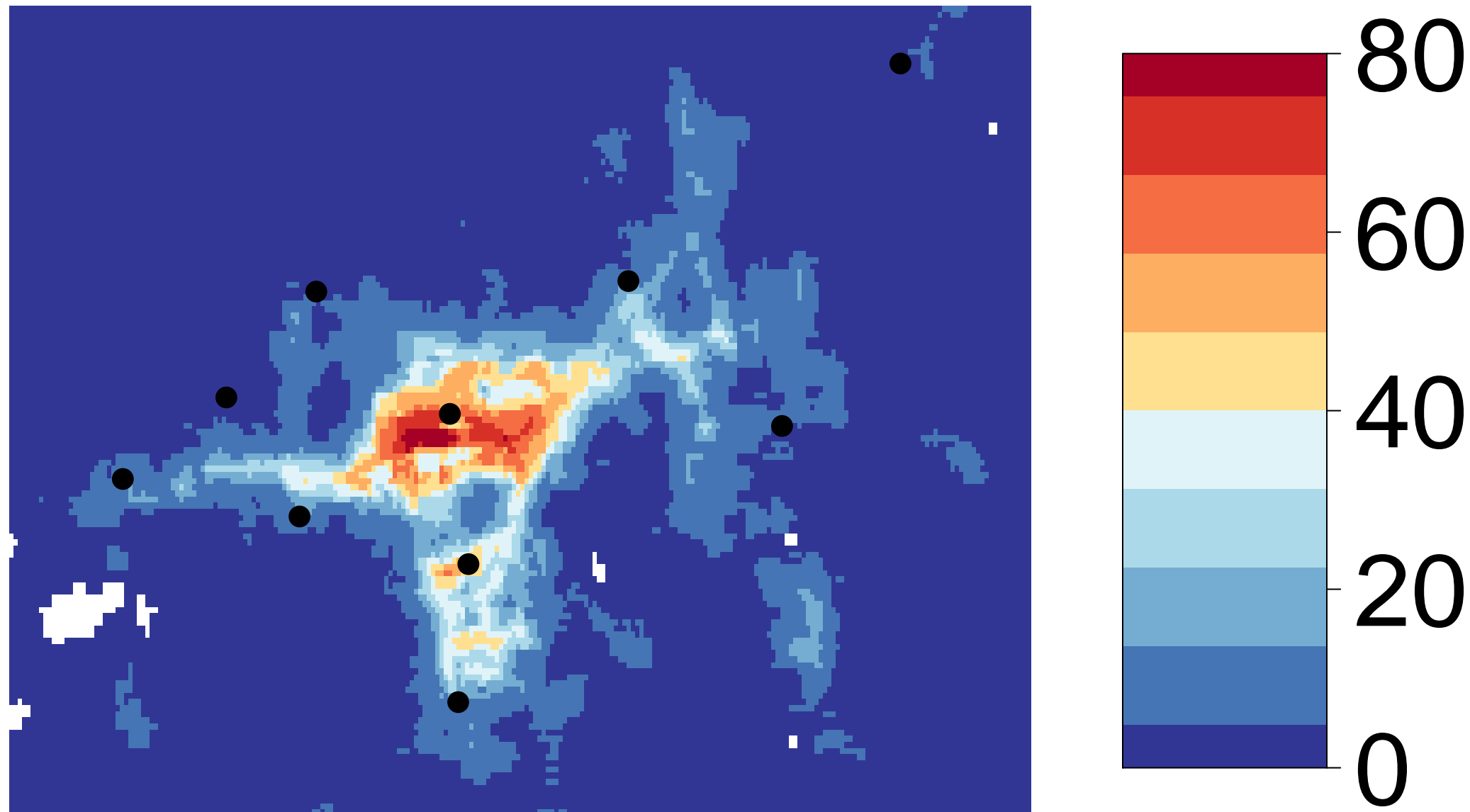

Supplement: Supplementary file 1 [file ijerph-17-02728-s001.zip › Figure S2.pdf]

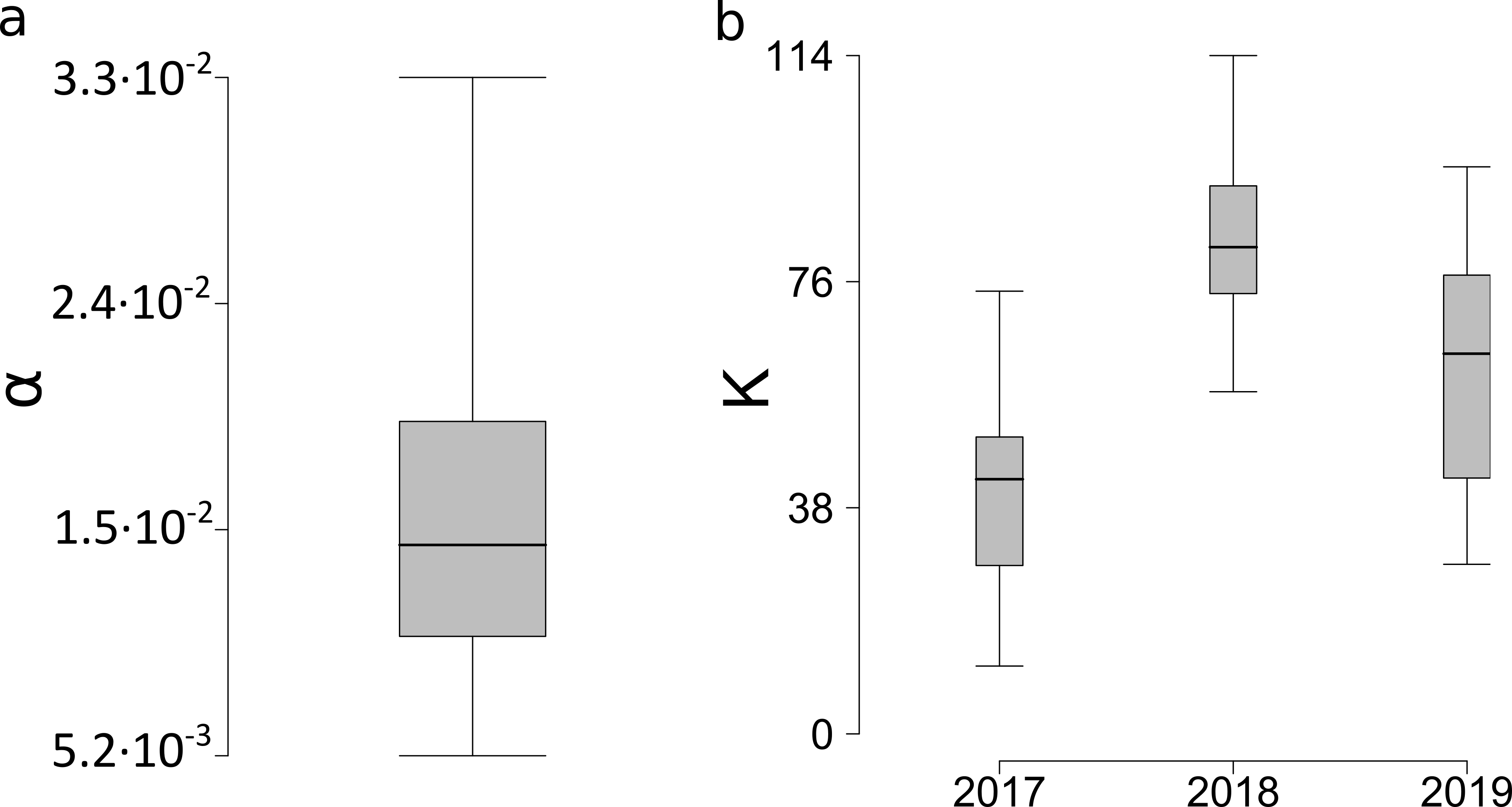

Supplement: Supplementary file 1 [file ijerph-17-02728-s001.zip › Figure S3.png]
